# Synergistic antitumor efficacy of aspirin plus lenvatinib in hepatocellular carcinoma via regulating of diverse signaling pathways

**DOI:** 10.1038/s41420-023-01664-y

**Published:** 2023-11-16

**Authors:** Xijing Yan, Haoyuan Yu, Jinliang Liang, Zhongying Hu, Xuejiao Li, Huanyi Liu, Jia Yao, Xin Sui, Jun Zheng, Rong Li

**Affiliations:** 1 Department of Hepatic Surgery and Liver Transplantation Center, Third Affiliated Hospital of Sun Yat-sen University, Guangzhou, 510630 China; 2 Guangdong Provincial Key Laboratory of Liver Disease Research, Third Affiliated Hospital of Sun Yat-sen University, Guangzhou, 510630 China; 3 Surgical ICU, Third Affiliated Hospital of Sun Yat-sen University, Guangzhou, 510630 China

**Keywords:** Drug development, Preclinical research

## Abstract

It has been established that monotherapy yields limited efficacy in treating hepatocellular carcinoma (HCC), especially advanced HCC. Increasing evidence from preclinical studies and clinical trials indicates that combining multiple drugs can potentially refine treatment efficacy. Accordingly, it is crucial to explore more effective clinically feasible combination therapies to enhance the treatment outcomes of HCC patients. This study evaluated the antitumor efficacy and safety of combination therapy involving aspirin and lenvatinib in HCC. Through in vitro and in vivo assays, we demonstrated that this combination yielded stronger antitumor effects compared to lenvatinib or aspirin monotherapy. Furthermore, no significant adverse events were observed in an HCC mouse model during treatment. Mechanistic studies revealed that aspirin plus lenvatinib could target multiple oncogenes and tumor suppressors, affecting diverse signaling pathways in various biological processes conducive to antitumor effects. Overall, our findings suggest that aspirin plus lenvatinib could serve as a promising combination regimen to improve the therapeutic outcomes of HCC.

## Introduction

Primary liver cancer is widely acknowledged as one of the leading causes of cancer-related death worldwide, ranking sixth in morbidity and third in mortality rates [1]. Hepatocellular carcinoma (HCC) is the most common type of primary liver cancer, accounting for up to 90% of cases, and its incidence has been steadily increasing in recent years [2]. Most HCC patients present with locally advanced or metastatic disease at clinical diagnosis, missing the optimal timing for surgical resection or liver transplantation [3]. During clinical practice, targeted therapies such as Sorafenib, Bevacizumab, Atezolizumab, and Lenvatinib are often indicated for patients with advanced HCC [4]. However, drug resistance and short duration of efficacy substantially limit the antitumor effect of targeted drugs [5, 6]. Increasing evidences suggest that combination medication will be the effective method for the treatment of advanced HCC in the future. Especially the positive results obtained from the clinical trials for lenvatinib plus pembrolizumab [7] further strengthen our confidence in investigating combination medications for advanced HCC.

Aspirin, a classical non-steroidal anti-inflammatory drug, has been widely used for the treatment of pain, fever and inflammatory disease [8]. In addition, aspirin is also used as an antiplatelet drug to prevent heart attacks and strokes [9, 10]. Recently, a series of randomized clinical trials and epidemiological studies suggest that regular use of aspirin can significantly reduce the incidence of several cancers, such as colon cancer, breast cancer and HCC [11–13]. Some preclinical studies also suggested that aspirin could exert antitumor effects in HCC [14]. Moreover, aspirin in combination with other antitumor drugs, such as sorafenib, doxorubicin, nutlin-3, 5-fluorouracil, and valproic acid, could achieve stronger antitumor effects than monotherapy [15, 16]. The above studies overlap in their assertion that aspirin can potentially be used in combination therapy for HCC.

Lenvatinib, a multi-target tyrosine kinase inhibitor (TKI), has been approved for the first-line treatment of patients with unresectable HCC in the USA, EU, Japan, and China since 2018. This approval marked a significant shift in the landscape of HCC treatment, as sorafenib had been the sole first-line TKI treatment for HCC for over a decade [17]. Lenvatinib has demonstrated an acceptable tolerability profile comparable to sorafenib, thereby facilitating its combination with other drugs for potential therapeutic strategies [18]. Positive outcomes have been documented in clinical trials for lenvatinib plus immune-checkpoint inhibitors (ICIs), such as pembrolizumab (an anti-PD-1 antibody), in the treatment of advanced HCC [7, 19]. However, due to the high heterogeneity of HCC, the efficacy of lenvatinib plus pembrolizumab is often inconsistent. Therefore, more efforts are needed to explore whether other drugs (especially those with proven clinical safety), when combined with lenvatinib, could achieve excellent antitumor efficacy in advanced HCC.

Herein, we investigated the antitumor effects of aspirin plus lenvatinib in HCC through a series of in vitro and in vivo assays for the first time. Our findings revealed that aspirin plus lenvatinib could achieve stronger antitumor efficacy in HCC compared to monotherapy. Furthermore, this combination did not cause significant adverse events, such as obvious weight loss, impaired liver/kidney function, or structural abnormalities of the liver, intestine, kidney, and spleen, and there were no incidents of gastrointestinal bleeding. We also evaluated the potential mechanism of the synergistic antitumor activity mediated by aspirin plus lenvatinib and substantiated that multiple oncogenes or tumor suppressors associated with proliferation (p-AKT, p-ERK, p-MEK, p21, p27, p-CDK2, p-Rb), metabolism (c-Myc, LDHA, p-AMPK, p-4EBP1), and immunity (COX2) were significantly regulated by the drug combination. Collectively, our findings suggest that aspirin plus lenvatinib could be used as a novel combination regimen in the treatment of advanced HCC.

## Results

### Aspirin significantly inhibits the growth of HCC cells in vitro

**Fig. 1:**
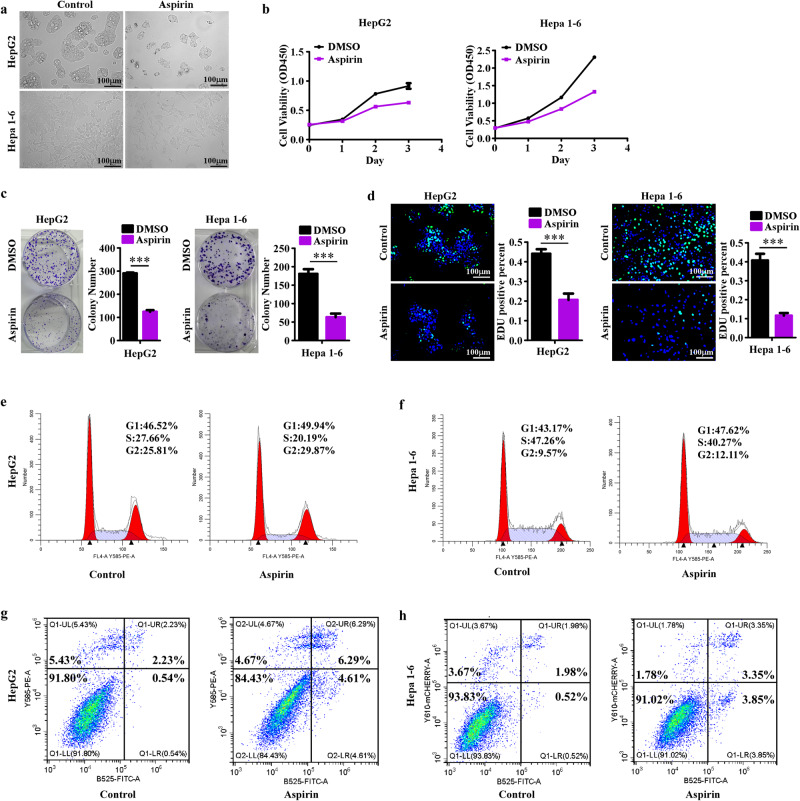
Aspirin inhibits the growth of HCC cells. **a** Representative images of HepG2 and Hepa1-6 cells treated with 4 mM aspirin and control solution for 48 h. **b** CCK8 assays reveal cell growth curves of HepG2 and Hepa1-6 cells treated with 4 mM aspirin and control solution. **c** Representative images (left) and relative quantification (right) for the colony formation assays of HepG2 and Hepa1-6 cells with different treatments. **d** Representative micrographs (left) and relative quantification (right) for the EdU assays of HepG2 and Hepa1-6 cells with different treatments. **e**, **f** Cell cycle detection assays display the growth inhibition of aspirin on HepG2 and Hepa1-6 cells. **g**, **h** Cell apoptosis detection assays show the apoptosis induction of aspirin on HepG2 and Hepa1-6 cells. Error bars represent the means of three independent experiments. *P < 0.05, **P < 0.01, ***P < 0.001.

Based on CCK8 assays, we determined the half maximal inhibitory concentration (IC50) value of aspirin in HepG2 and Hepa1-6 (Supplementary Fig.1a, b). To assess the antitumor efficiency of aspirin on HCC, we observed the morphological changes in HepG2 and Hepa1-6 cells treated with aspirin. As shown in Fig. 1a, the cell abundance in the aspirin group was significantly lower than in the control group, and the morphology of HepG2 and Hepa1-6 cells was relatively flatter in the aspirin group. Then, we detected the effect of aspirin on cell viability via CCK8 assays. As shown in Fig. 1b, aspirin significantly decreased cell viability. We also performed colony formation assays (Fig. 1c) and demonstrated the strong inhibitory effect of aspirin on the colony formation abilities of HepG2 and Hepa1-6 cells. The decline in cell viability and colony formation could be attributed to multiple reasons, including decreased proliferation, increased apoptosis, or both. EdU and cell cycle detection assays revealed that aspirin could strongly downregulate the EdU-positive percentage and reduce the proportion of cells in the S phase (Fig. 1d–f). Then, the cell apoptosis detection assays (Fig. 1g, h) showed that aspirin also triggered slight induction of cell death. Taken together, these findings suggested aspirin could effectively inhibit cell proliferation and slightly induce cell apoptosis.

### Aspirin regulates the expression of multiple oncogenes and tumor suppressors

**Fig. 2:**
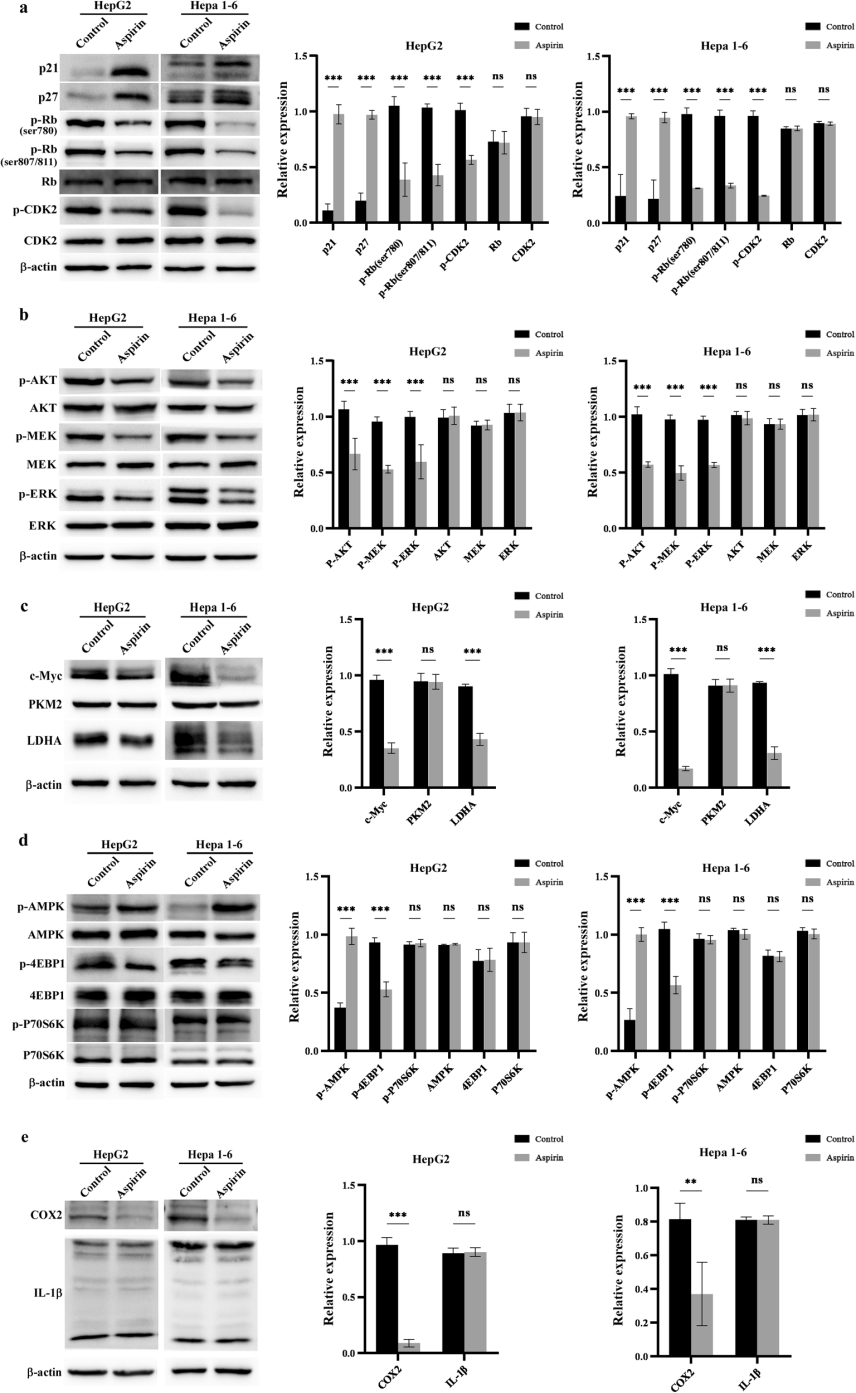
Aspirin regulates the expression and phosphorylation of multiple molecules related to tumor progression. Cells were treated with 4 mM aspirin or control solution for 48 h, then harvested for subsequent western blotting. **a** Effects of aspirin on the expression of proteins associated with the cell cycle (P21, P27, and the phosphorylation of Rb and CDK2). **b** Effects of aspirin on the phosphorylation of AKT, MEK, and ERK, which regulated the activities of Ras/Raf/MAPK and PI3K/AKT pathways. **c** Effects of aspirin on the expression of proteins related to cell metabolism (c-Myc, PKM2 and LDHA). **d** Effects of aspirin on the phosphorylation of AMPK, 4EBP1 and P70S6K, which related to the activity of AMPK/mTOR pathway. **e** Effects of aspirin on the expression of COX2 and IL-1β, which related to immunoregulation. Error bars represent the means of three independent experiments. *P < 0.05, **P < 0.01, ***P < 0.001, NS no significance.

To reveal the mechanism underlying the antitumor effects of aspirin against HCC, we first examined the effects of aspirin on cell cycle-related regulatory proteins. As shown in Fig. 2a, aspirin could upregulate the expression of P21 and P27 and inhibit the phosphorylation of Rb and CDK2. Given that the overactivation of the PI3K/AKT and MAPK/ERK pathways plays important roles in HCC growth, we examined the effect of aspirin on them. As shown in Fig. 2b, aspirin could significantly decrease the phosphorylation of AKT, MEK, and ERK. Previous studies also showed that aspirin could regulate cell metabolism, such as glycolysis, to inhibit tumor progression. As shown in Fig. 2c, aspirin could reduce the expression of c-Myc and LDHA, while PKM2 levels were unaffected. Then, we examined the effects of aspirin on AMPK, COX2, and IL-1β, other classic targets of aspirin. As shown in Fig. 2d, aspirin significantly promoted the phosphorylation of AMPK and inhibited the phosphorylation of 4EBP1, while P70S6K expression was unaffected. COX2 and IL-1β are mainly involved in immune regulation. In our study, we found that aspirin could decrease the expression of COX2, consistent with the literature, while IL-1β expression experienced no significant change (Fig. 2e). Taken together, these findings suggested aspirin can regulate the expression of multiple oncogenes and tumor suppressors, thereby influencing diverse signaling pathways to exert powerful antitumor effects. The diverse and multi-targeted characteristics of aspirin and its relative clinical safety make it a suitable candidate for drug combinations in treating HCC.

### Aspirin plus lenvatinib yield a robust antitumor efficiency in HCC in vitro

Synergistic effects occur when two or more drugs are used together, resulting in a greater overall effect than the sum of the effects achieved from monotherapy. This drug combination can enhance efficacy, reduce drug dosage, and minimize potential side effects. After establishing the antitumor effects of aspirin in HCC, we sought to investigate whether the combination of aspirin and lenvatinib yielded synergistic antitumor effects. To address this, we performed a series of in vitro assays. The IC50 values of lenvatinib in HepG2 and Hepa1-6 cells (Supplementary Fig. 2a, b) were detected using CCK8 assays. We first conducted drug synergy studies based on the IC50 values of aspirin (Supplementary Fig. 1a, b) and lenvatinib in HCC cells. The combination indices (CI) for lenvatinib and aspirin were subsequently calculated and shown in Table 1. CI value < 0.9 indicates a synergistic effect between the two drugs. The detailed CI values in Table 1 showed that aspirin plus lenvatinib could exert a synergistic antitumor effect on HCC cells at different concentrations.

**Table 1:** Combination indices (CI) for aspirin and lenvatinib.

Cell Line	Lenvatinib (μM)	Aspirin (mM)	CI	Synergy
HepG2	5	5.959	0.895	+
	10	3.325	0.761	++
	15	1.449	0.717	++
Hepa1-6	5	4.957	0.873	+
	10	2.767	0.758	++
	15	1.288	0.741	++

Based on the detailed CI values, the synergy strength is displayed with graded symbols. “+” indicates slight synergism; “++” indicates moderate synergism; “+++” indicates strong synergism.

**Fig. 3:**
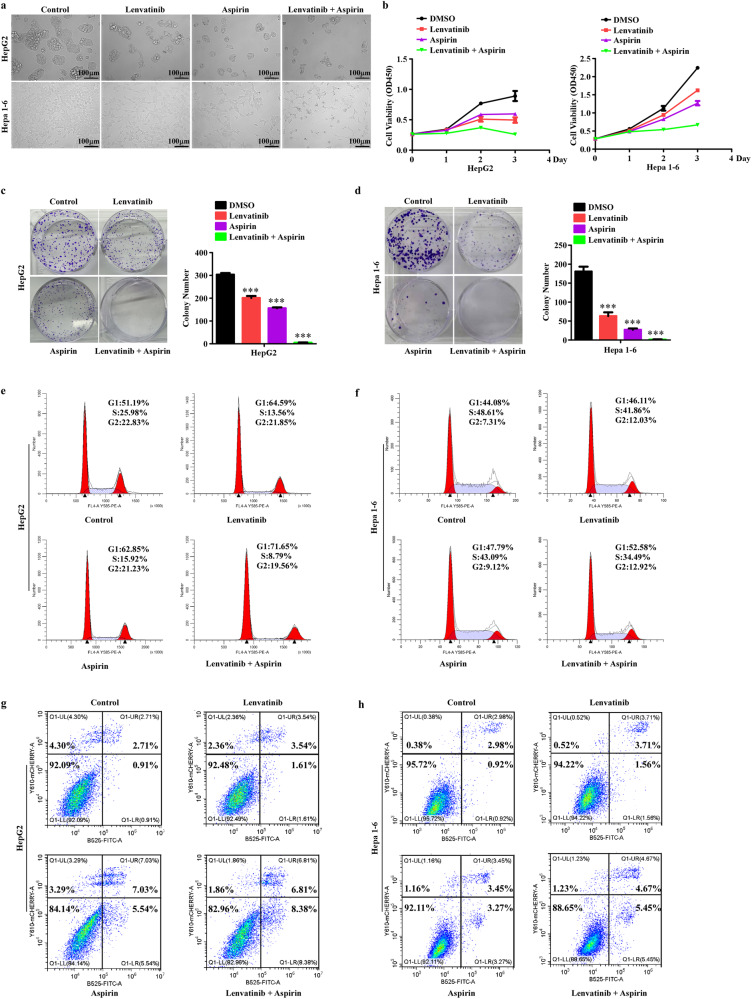
Aspirin plus lenvatinib exert stronger antitumor effects in HCC cells than monotherapy. **a** Representative images of HepG2 and Hepa1-6 cells treated with different drugs. **b** CCK8 assays reveal cell growth curves of HepG2 and Hepa1-6 cells treated with different drugs. **c**, **d** Representative images (left) and relative quantification (right) for the colony formation assays of HepG2 (**c**) and Hepa1-6 (**d**) cells treated with different drugs. **e**, **f** Cell cycle detection assays analyze the effects mediated by different drugs on the proliferation of HepG2 (**e**) and Hepa1-6 (**f**) cells. **g**, **h** Cell apoptosis detection assays reveal the effects of different drugs on apoptosis induction in HepG2 (**g**) and Hepa1-6 (**h**) cells. Error bars represent the means of three independent experiments. *P < 0.05, **P < 0.01, ***P < 0.001. Aspirin, 4 mM; lenvatinib, 10 μM.

Then, we observed the morphological changes in HepG2 and Hepa1-6 cells treated with aspirin plus lenvatinib. Compared with monotherapy (aspirin or lenvatinib), combination therapy was associated with decreased cell abundance and worse cellular state, with most cells undergoing serious shrinkage, especially Hepa1-6 cells (Fig. 3a). Moreover, CCK8, colony formation, EdU, Cell Cycle Detection, and Apoptosis Detection assays were performed to detect the antitumor effects of aspirin plus lenvatinib. As shown in Fig. 3b–f and Supplementary Fig. 2c, d, combination therapy could further inhibit cell viability, colony formation ability, and cell proliferation ability and promote cell cycle arrest. Aspirin plus lenvatinib yielded robust growth inhibition resulting in a near cessation of HCC cell proliferation. In addition, the results in Fig. 3g, h showed that unlike sorafenib, lenvatinib nearly did not induce cell apoptosis, while aspirin plus lenvatinib showed slightly higher cell apoptosis than that of aspirin alone. Collectively, these results strongly suggest that the combination of aspirin and lenvatinib exerts a robust synergistic antitumor activity in HCC cells in vitro.

### Identification of key molecules regulated by aspirin plus lenvatinib

**Fig. 4:**
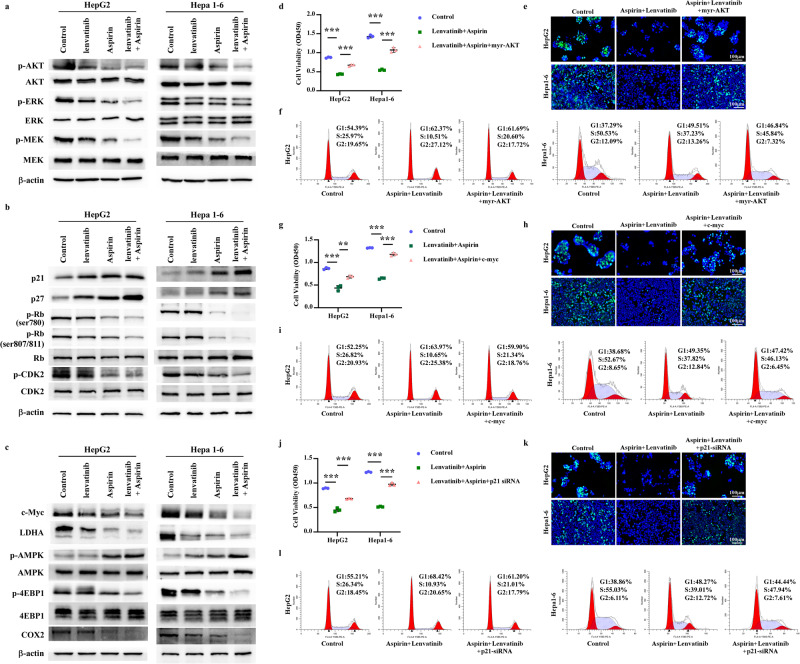
The antitumor efficacy of aspirin plus lenvatinib depends on its regulation of multiple oncogenes and tumor suppressors. **a**–**c** HepG2 and Hepa1-6 Cells were separately treated with control solution, 4 mM aspirin, 10 μM lenvatinib, or aspirin (4 mM) plus lenvatinib (10 μM) for 48 h. Western Blotting assays were used to detect the effects of different drugs on **a** the phosphorylation of AKT, MEK, and ERK, which regulated the activities of Ras/Raf/MAPK and PI3K/AKT pathways; **b** the expression of proteins associated with the cell cycle (P21, P27 and the phosphorylation of Rb and CDK2); **c** the expression of proteins related to cell metabolism (c-Myc, LDHA), the activity of AMPK/mTOR pathway (the phosphorylation of AMPK, 4EBP1) and immunoregulation (COX2). **d** CCK8 assays reveal the effect of AKT activation on cell viability of HCC cells mediated by the drug combination. **e** Representative micrographs for the EdU assays show the effect of AKT activation on cell proliferation of HCC cells mediated by the drug combination. **f** Cell cycle detection assays display the effect of AKT activation on the growth inhibition of drug combination on HepG2 and Hepa1-6 cells. **g**–**i** CCK8 assays (**g**), EdU assays (**h**), and cell cycle detection assays (**i**) show the effect of c-Myc overexpression on the decrease in cell viability, growth inhibition and cell cycle arrest induced by aspirin plus lenvatinib. **j**–**l** CCK8 assays (**j**), EdU assays (**k**), and cell cycle detection assays (**l**) reveal the effect of p21 silencing on the antitumor efficacy mediated by aspirin plus lenvatinib. Error bars represent the means of three independent experiments. *P < 0.05, **P < 0.01, ***P < 0.001.

It is well-established that lenvatinib can target various molecules, including vascular endothelial growth factor receptor (VEGFR) 1-3, fibroblast growth factor receptor (FGFR) 1-4, platelet-derived growth factor receptor (PDGFR) α, as well as proto-oncogenes RET and KIT [20]. The phosphorylation levels of AKT, ERK, and MEK indicate lenvatinib’s antitumor effect to some extent. Building on the targets regulated by aspirin in Fig. 2, we further investigated the potential antitumor mechanisms of aspirin plus lenvatinib. As shown in Fig. 4a–c, aspirin plus lenvatinib could further decrease the phosphorylation of AKT, MEK, and ERK compared to monotherapy. Lenvatinib yielded little or no inhibitory effect on the phosphorylation of Rb and CDK2, while drug combination could substantially decrease phosphorylation levels. The drug combination could also enhance the expression of p21 and p27 and reduce the expression of c-Myc and LDHA compared to monotherapy. Then, we examined the effect of aspirin plus lenvatinib on AMPK. We found that lenvatinib had no or slight increase on the phosphorylation of AMPK, while the p-4EBP1 were significantly inhibited by drug combination than aspirin or lenvatinib alone, which may be due to that the phosphorylation of 4EBP1 can be regulated by other molecules besides AMPK. In addition, we also assessed the regulatory effect of the drug combination on COX2 expression, and found that lenvatinib had no or slightly inhibition on the expression of COX2, while the combination of drugs significantly decreased COX2 levels compared to monotherapy. The relative protein quantification of these oncogenes and tumor suppressors was showed in Supplementary Fig. 3a–c.

To further validate the roles of these oncogenes or suppressors in the antitumor efficacy mediated by aspirin plus lenvatinib, we performed corresponding functional complement experiments. Based on the functions of these molecules in tumors and the changes in their protein levels during drug combination, we selected AKT, c-Myc, and p21 as representatives for functional complement experiments. Using CCK8, EdU, and cell cycle detection assays, we evaluated the influence of AKT activation, c-Myc overexpression, and p21 silencing on the antitumor efficacy of aspirin plus lenvatinib. As shown in Fig. 4d–f and Supplementary Fig. 3d, overexpression of constitutionally activated AKT (myristoylated AKT or myr-AKT) significantly reversed the decrease in cell viability and proliferation and alleviated cell cycle arrest induced by the drug combination. Similar to constitutive AKT activation, overexpression of c-Myc or silencing of p21 strongly weakened the antitumor efficacy mediated by aspirin plus lenvatinib (Fig. 4g–l, Supplementary Figs. 3eand 4f). In addition, we observed that altering the activity or expression of a single oncogene or suppressor alone did not fully reverse the antitumor effects of aspirin plus lenvatinib in HCC cells. This suggests that the combined drug efficacy relies on the coordinated regulation of multiple oncogenes (such as AKT and c-Myc) and tumor suppressors (such as p21).

Taken together, our findings suggest that aspirin plus lenvatinib significantly potentiates the regulation of multiple oncogenes and tumor suppressors compared to monotherapy, thereby exerting stronger synergistic antitumor effects against HCC cells.

### Aspirin plus lenvatinib significantly inhibits the proliferation and tumor angiogenesis of HCC in vivo

Based on the above results, we further evaluated the antitumor efficacy of aspirin plus lenvatinib in an HCC mouse model. We subcutaneously implanted Hepa1-6 cells (1 × 10^6^) on the inguinal fold of each C57BL/6 J mice. When most tumors size reached 100 mm^3^, all mice were randomly divided into four groups to receive treatment of PBS, aspirin (100 mg/kg/day, i.g.), lenvatinib (20 mg/kg/day, i.p.) and aspirin (100 mg/kg/day, i.g.) plus lenvatinib (20 mg/kg/day, i.p.) for 20 days. Tumor size and mouse weight were measured every two days. And the appetite and dynamics of mice were observed daily.

**Fig. 5:**
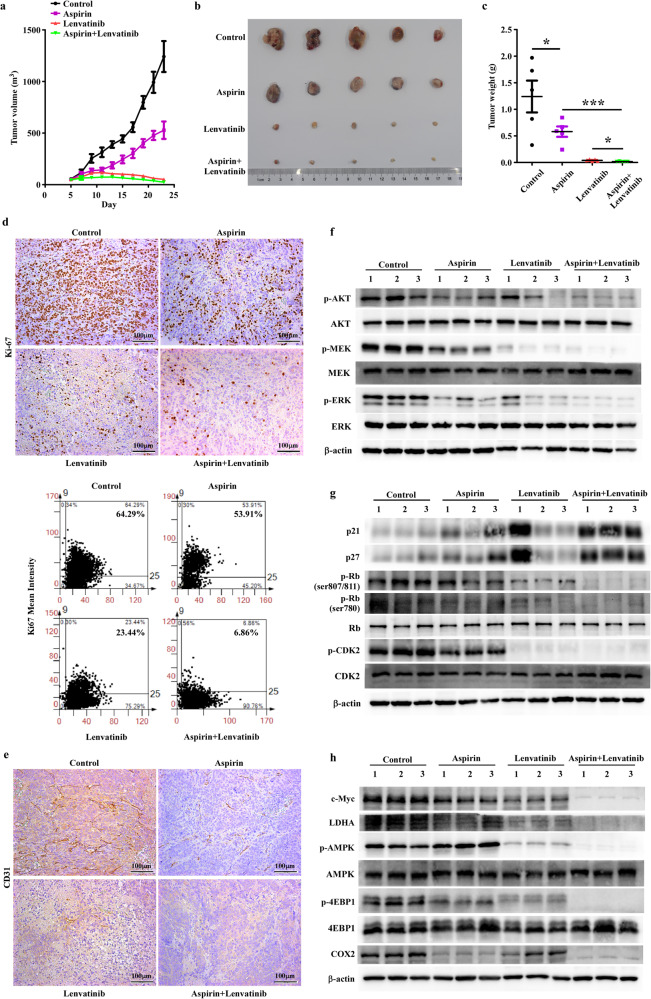
Aspirin plus lenvatinib significantly inhibits HCC tumor growth in vivo. **a** Growth curves indicating the average tumor volume at each time point in cohorts of mice with different treatments. **b**, **c** At the experimental endpoint, tumors were dissected, then photographed (**b**) and weighted (**c**) as indicated. **d** Representative images (upper) and relative quantification (lower) for Ki-67 staining reveal the effects of different drugs on the proliferation of HCC tumors. **e** CD31 staining reveals the effects of different drugs on the angiogenesis of HCC tumors. **f**–**h** Western Blotting assays analyze the regulation of different drugs on (**f**) the phosphorylation of AKT, MEK, and ERK, which regulated the activities of Ras/Raf/MAPK and PI3K/AKT pathways; **g** the expression of proteins associated with the cell cycle (p21, p27 and the phosphorylation of Rb and CDK2); **h** the expression of proteins related to cell metabolism (c-Myc, LDHA), the activity of AMPK/mTOR pathway (the phosphorylation of AMPK, 4EBP1) and immunoregulation (COX2). For **a** and **c**, data are presented as mean ± SD (*n* = 5 mice/group).

The tumor growth curve (Fig. 5a) showed that aspirin or lenvatinib monotherapy could significantly decrease tumor growth rate compared to the control group, with a more pronounced effect observed for lenvatinib. As expected, the drug combination exhibited a more significant inhibitory effect on tumor growth than monotherapy. Then, we measured the size and weight of dissected tumors. As shown in Fig. 5b, c, the size and weight of the tumors were significantly reduced after treatment with aspirin, lenvatinib, and the drug combination. Among them, aspirin plus lenvatinib yielded the best effect consistent with the results in Fig. 5a. We also performed ki-67 (Fig. 5d-upper) and CD31(Fig. 5e) staining to prove that drug combination could enhance the inhibition of tumor proliferation and angiogenesis compared to monotherapy. Based on the results of Fig. 4a–c, we further examined the regulation effects of drug combination on these molecules with mice tumor tissues (Fig. 5f–h). Relatively, the synergistic antitumor efficacy of aspirin plus lenvatinib in vivo seemed to be weaker than that in vitro. We supposed that these may be attributed to the dosage of lenvatinib we used for the in vivo experiments in this study. In the past, both Laura Torrens et al. [21]. and Chenhe Yi et al. [22]. detected the antitumor effect of lenvatinib on HCC mouse model with the dosage of 10 mg/kg, while we herein applied 20 mg/kg. To more unequivocally reflect the synergistic antitumor efficacy of aspirin plus lenvatinib in vivo, the results of ki-67 staining (Fig. 5d-upper) and WB assays (Fig. 5f–h) were further analyzed and the relative quantification results were showed in Fig. 5d-lower and Supplementary Fig. 4a–c.

Collectively, these results substantiated that aspirin plus lenvatinib yielded a stronger antitumor effect than monotherapy by collaboratively regulating the expression of multiple oncogenes and tumor suppressors in an HCC mouse model.

### Safety assessment of aspirin plus lenvatinib

Although the clinical safety of aspirin and lenvatinib has been fully evaluated, it remains unclear whether their combination will cause adverse events. In addition, several studies demonstrated that aspirin could increase the risk of gastrointestinal bleeding. Accordingly, we evaluated the influence of different drugs on body weight, liver/kidney function, and the histological morphology of the liver, intestine, spleen, and kidney with a mouse model. As shown in Supplementary Fig. 5a, the body weight of four group mice gradually increased, and drug treatment did not cause drastic changes in body weight. At the endpoint of the animal experiments, we collected the blood of mice and examined their liver and kidney function. As shown in Supplementary Fig. 5b, drug treatment could cause a slight elevation of AST/ALT and these mild changes did not cause liver damage. The levels of BUN and Cre in serum did not increase, even slightly decreased, after drug treatment (Supplementary Fig. 5c). Then, we performed HE staining to evaluate whether drug treatment could cause structural lesions of the liver, intestine, kidney, and spleen. As shown in Supplementary Fig. 5d, drug treatment did not cause significant histological morphological changes. Especially in intestine, the structure of small intestine villi was normal and there was no intestinal bleeding. In addition, we observed the effects of drugs on the appetite and dynamics of mice, and no obvious abnormalities were found. Taken together, our in vivo results demonstrated that both monotherapy and drug combination were relatively safe, and no obvious adverse events occurred during treatment.

## Discussion

Chemotherapy remains the mainstay of treatment for patients with advanced HCC. Unfortunately, the heterogeneity of HCC and its propensity to develop chemoresistance pose significant challenges. In recent years, combination therapy has emerged as a promising strategy for treating advanced HCC. Preclinical and clinical studies have demonstrated positive outcomes for combination therapies, indicating that they may become the mainstay of treatment for this condition in the future. Some successful combination regimens include lenvatinib plus pembrolizumab and bevacizumab plus atezolizumab have yielded positive results, and these two combination regimens are now being used in clinical practice [7, 23]. In addition, several other combination therapies are being investigated in preclinical and clinical trials [24]. Due to the highly heterogeneous nature of HCC, there is an urgent need to develop more drug combinations that are highly effective and clinically feasible.

Herein, we proved that aspirin plus lenvatinib yielded superior antitumor efficacy against HCC compared to monotherapy for the first time, which is worth to promote them in performing subsequent clinical trials. However, before clinical application, it is noticed that high toxicity is a common cause of failure in preclinical and clinical trials for drug combinations. The most common adverse events caused by lenvatinib include hypertension, decreased appetite, proteinuria, fatigue and weight loss [25]. And high dose of aspirin can increase the risk of gastrointestinal bleeding [26]. Therefore, we carefully assessed the toxicity effects of aspirin plus lenvatinib in terms of body weight, appetite, behavior, liver/kidney function, and the morphology of liver, intestine, kidney, and spleen tissues in the mice of the treatment groups. And we did not observed significant adverse events occurred in both monotherapy and combination groups, indicating that aspirin plus lenvatinib is relatively safe. Although these, patients with severe portal hypertension or coagulation disorders as well as having risk of gastrointestinal bleeding still prudent to plus aspirin during lenvatinib treatment.

In addition to evaluating the antitumor effects of aspirin plus lenvatinib, we investigated the underlying mechanisms. Previous studies have suggested that aspirin can regulate various processes in cancer cells, such as proliferation, apoptosis, metabolism, and immunity [27]. In this study, we conducted a comprehensive analysis of the regulatory effects of aspirin on these processes and found that aspirin significantly influenced the expression of p21, p27, c-Myc, LDHA, COX2, and the phosphorylation of CDK2, Rb, AKT, ERK, MEK, AMPK, and 4EBP1. It’s worth noting that we did not observe aspirin had a strong effect on cell apoptosis. Considering drug combination, we also evaluated the effects of lenvatinib on the targets of aspirin. The in vitro results showed that lenvatinib could significantly regulate the expression of c-Myc, LDHA, p21 and p27, while with no or slightly effects on the phosphorylation of Rb, CDK2 and 4EBP1. Interestingly, aspirin plus lenvatinib exhibited a stronger regulatory effect on these molecules than aspirin alone, indicating a synergistic effect in regulating multiple oncogenes and tumor suppressors. This synergistic effect was further validated in a mouse model of HCC.

AMPK is another important target of aspirin [28], we observed that the effect of lenvatinib on the phosphorylation of AMPK was inconsistent in in vitro and in vivo assays. Previous studies suggested that AMPK may be a context-dependent tumor suppressor or oncogene. On one hand, AMPK inhibits the activation of mTORC1 to exert antitumor effects. On the other hand, AMPK can active cAMP-PKA-CREB/ATF1 signaling to reprogram energy metabolism and help cells tolerate energy stress [29]. We speculated that HCC cells in the in vitro culture were not under significant energy stress, and AMPK mainly acted as a tumor suppressor. However, in the HCC mouse model, the shortage of nutrients induced AMPK to exert tumor protection, thereby weakening the antitumor effects of aspirin. The significantly inhibition of AMPK by lenvatinib in mouse model may enhance the sensitivity of HCC cells to aspirin, while the underline mechanisms is worthy of further investigation.

In recent years, research on the combination of lenvatinib with immune-checkpoint inhibitors (ICIs), such as pembrolizumab, has gained significant momentum. Previous studies have shown that lenvatinib yields immunomodulatory capabilities by inhibiting VEGFR, leading to increased activity of CD8 + T cells and reduced infiltration of tumor-associated macrophages (TAMs) [17, 21]. However, HCC is considered a “cold” tumor, with limited immune cell infiltration and most immune cells in a state of exhaustion, which limits the efficacy of lenvatinib plus pembrolizumab for patients. Therefore, finding ways to effectively transform “cold” tumors into “hot” tumors is an important area of exploration. COX2 is widely acknowledged as a direct target of aspirin and has been linked to immunosuppression [30]. PGE2, the catalyzed production of COX2, could induces the expansion of myeloid-derived suppressor cells (MDSC), which inhibit the antitumor effects of cytotoxic T lymphocyte and increasing regulatory T Cell (Treg) and regulatory dendritic cell responses in the tumor microenvironment [31]. Moreover, inhibition of COX2 by aspirin could also recruit natural killer cells and cytotoxic T lymphocytes into tumor microenvironment via increasing the secretion of CXCL9 and CXCL10 [32]. The antitumor effects of aspirin combined with anti-PD-L1 blockade have been evaluated in mouse models, and an ongoing clinical trial (NCT 02659384) in Switzerland is assessing the effects of aspirin plus anti-PD-L1 antibodies (atezolizumab, bevacizumab) in ovarian cancer. In this study, we also demonstrated that aspirin plus lenvatinib could significantly inhibit the expression of COX2 in HCC compared to monotherapy. Considering the manageable toxicity of aspirin plus lenvatinib in the mouse model and the potential immunomodulatory abilities of both drugs, we speculate that their combination may enhance the effects of subsequent immunotherapy on HCC, which is worthy of further research.

In conclusion, we evaluated the antitumor effects and drug safety of aspirin plus lenvatinib in HCC for the first time. Additionally, we investigated the potential mechanisms through which aspirin plus lenvatinib functions in HCC and demonstrated that this combination could target multiple oncogenes and tumor suppressors to collaboratively inhibit tumor progression. Furthermore, as aspirin and lenvatinib have been used in clinical practice for many years, their combination facilitates the subsequent design of clinical trials and translation into real-world applications.

## Materials and methods

### Cell culture

HCC cell lines HepG2 (homo sapiens) and Hepa1-6 (Mus musculus) were purchased from the Cell Bank of Type Culture Collection (Shanghai City, China). Both cell lines were cultured in RPMI-1640 medium (Invitrogen, Carlsbad, CA) supplemented with 10% FBS (Gibco, Carlsbad, CA, USA) and 1% penicillin/streptomycin (Invitrogen) at 37 °C in a humidified air atmosphere containing 5% carbon dioxide. Both cell lines used in this study have been authenticated within the last one year using STR profiling. All experiments were performed with cells free of mycoplasma contamination.

### CCK8 assays

HepG2 and Hepa1-6 cells were seeded in 96-well plates (3000 cells/well), then treated with different drugs, The count of viable cells was measured using a detection kit purchased from ESscience (ES7011, Shanghai City, China). A spectrophotometer was used to measure the absorbance at 450 nm. Three parallel replicates were set for each group.

### Colony formation assays

HepG2 (800 cells/well) and Hepa1-6 cells (600 cells/well) were seeded in 6-well plates. The related drugs were added to the plates on day 4 and cells were cultured for another 6 (Hepa1-6) or 10 days (HepG2). Then, the colonies were washed with PBS, fixed with 4% paraformaldehyde and stained with 0.1% crystal violet at room temperature for 30 min. Colonies were counted using Image-Pro Plus 6.0. The experiments were independently performed in triplicate.

### EdU assays

HepG2 and Hepa1-6 cells were seeded in 24-well plates (30,000 cells/well), then treated with different drugs for 48 h. The subsequent EdU staining were performed with a key Fluor488-EdU kit (KGA331, KeyGEN Bio TECH, Jiangsu, China) according to the manufacturer’s protocol. Images were obtained using an inverted fluorescence microscope (Carl Zeiss, Jena, Germany). The proportion of EdU-positive cells was determined as: EDU-positive cells/DAPI-positive cells. The experiments were independently performed in triplicate.

### Cell cycle detection assays

HepG2 and Hepa1-6 cells were seeded in 6-well plates (250 000 cells/well), and treated with different drugs for 48 h. The cell cycle detection kit was purchased from KeyGEN Bio TECH (KGA512, Jiangsu, China). Cell samples were processed according to the manufacturer’s protocol, and the proportions of cells in different phases were measured using CytoFLEX LX (Beckman Colter, Inc, CA. USA). Next, the data were collected and processed using ModFit LT 4.1 (Verity Software House, USA).

### Cell apoptosis detection assays

HepG2 and Hepa1-6 cells were seeded in 6-well plates (250,000 cells/well) and treated with different drugs for 48 h. Annexin V-FITC apoptosis detection kits (KGA108, KeyGEN Bio TECH) were purchased for the preparation of cell samples, and the subsequent measure was performed using CytoFLEX LX (Beckman Colter, Inc, CA. USA). Next, the data were collected and processed using CytExpert 2.0 (Beckman Colter, Inc, CA. USA).

### Western blotting

Western blotting was performed according to a previously described standard method using anti-1L-1β (ab254360, 1:1000; Abcam), anti-COX2 (#12282, 1:500; CST), anti-c-Myc (#9402, 1:1000; CST), anti-PKM2 (#4053, 1:1000; CST), anti-LDHA (#2012, 1:1000; CST), anti-p21 (#37543, 1:500; CST), anti-p27 (#2552, 1:500; CST), anti-p-Rb (ser780) (#8180, 1:1000; CST), anti-p-Rb (ser807/811) (#8516, 1:1000; CST), anti-p-CDK2 (#2561, 1:500; CST), anti-p-AMPK (#50081, 1:1000; CST), anti-p-4EBP1 (#2855, 1:1000; CST), anti-p-p70S6K (#9208, 1:1000; CST), anti-p-AKT (ser473) (#4060, 1:1000; CST), anti-p-MEK (#9154, 1:1000; CST), anti-p-ERK (#4370, 1:1000; CST), anti-AKT (#4691, 1:1000; CST), anti-p70S6K (#9202, 1:1000; CST), anti-AMPK (#5831, 1:1000; CST), anti-Rb (#9313, 1:1000; CST), anti-CDK2 (#2546, 1:1000; CST), anti-4EBP1 (#9644, 1:1000; CST), anti-MEK (#4694, 1:1000; CST), anti-ERK (#4695, 1:1000; CST), anti-β-actin (#3700, 1:2000; CST) antibodies. The experiments were independently performed in triplicate.

### Drug synergy experiments

Based on the IC50 values of aspirin and lenvatinib in HepG2 and Hepa1-6 cell lines, we chose a range of aspirin concentrations (2 mM, 4 mM, 6 mM) combined with three fixed concentrations of lenvatinib (5 μM, 10 μM, 15 μM) to conduct drug synergy experiments. HepG2 and Hepa1-6 cells were exposed to different dose combinations of these drugs for 48 h, and cell viability was subsequently assessed using the CCK8 assay. All experiments were repeated in triplicate, and a best fit trend-line was used to determine the corresponding IC50 values of aspirin for each of the 3 fixed doses of lenvatinib.

Combination index (CI) values were used to evaluate synergism between aspirin and lenvatinib. The CI was calculated using the Chou-Talalay equation: CI = a/Ai + b/Bi where a and b indicate the concentrations of drug A and drug B utilized in combination necessary to reduce cell viability by 50%, while Ai and Bi indicate the IC50 values of each drug used individually. CI > 1.1 indicates the combination of two drugs is antagonistic, 1.1 < CI < 0.9 indicates the combination of two drugs is additive, and CI < 0.9 indicates the combination of two drugs is synergistic [33].

### Plasmids, siRNAs and transfection

Human c-Myc plasmid (OENM_002467-2) was purchased from DHbio (Guangzhou, China) and mouse c-Myc plasmid (P0939) was purchased from MIAOLING BIOLOGY (Wuhan, China). The myr-AKT plasmids were obtained from Mengfeng Li’s laboratory at Sun Yat-sen University. The siRNAs of p21 were generated by GenePharma (Shanghai, China). The transfection of plasmids and siRNAs was performed using jetPRIME Transfection Reagent (101000046, Polyplus-transfection, France) according to the manufacturer’s instructions. The siRNA sequences of p21 were as follows: Human-p21-siRNA: CCAAACGCCGGCTGATCTT; Mouse-p21-siRNA: CCAAGCGCAGATTGGTCTT; Negative control: TTCTCCGAACGAGTCACGT.

### Subcutaneous tumor model of mice

For the in vivo experiments, twenty 6-week-old C57BL/6 J male mice were purchased. 1 × 10^6^ Hepa1-6 cells were subcutaneously injected into the inguinal fold of each C57BL/6 J mice. After 5 days, when the volume of most tumors reached 100 mm^3^, the mice we randomly divided into four groups: control, aspirin (S3017, Selleck, Houston, TX, USA), lenvatinib (S1164, Selleck, Houston, TX, USA) and aspirin plus lenvatinib (*n* = 5, per group) based on tumor size. The related drugs were administered as follows: In the aspirin group, aspirin was administered at a dosage of 100 mg/kg/day via intragastric (i.g.) administration. In the lenvatinib group: lenvatinib was administered at a dosage of 20 mg/kg/day via intraperitoneal (i.p.) injection. In the aspirin plus lenvatinib group, aspirin (100 mg/kg/day, i.g.) was followed by lenvatinib (20 mg/kg/day, i.p.) with a half-hour interval between administrations. In the control group, all mice were given equal volume of PBS every day. The drugs were administered for 20 days. The mice were monitored daily, and their appetite and behavior were observed. Tumor size and mouse weight were measured every two days. At the end of the experiment, all mice were anesthetized and euthanized, and the blood, liver, intestine, kidney, spleen, and tumors were collected for further analysis.

### Immunohistochemistry (IHC) and hematoxylin-eosin staining (HE)

IHC were performed according to a standard methods previously described, using anti-Ki-67 (GB111499, 1:400, Servicebio, Wuhan, China) and anti-CD31 (GB11063, 1:200, Servicebio, Wuhan, China). HE were also performed according to a standard methods previously described. Images were obtained using an upright fluorescence microscope (Carl Zeiss, Jena, Germany), and 5 visual fields were randomly taken for each slide.

### Assessment of liver/kidney function

At the endpoint of animal experiments, we collected the blood of mice and extracted the serum. The serum levels of ALT and AST were examined to assess liver function, and the serum levels of BUN and Cre were examined to reflect kidney function. The analyses and measurements were conducted at the Department of Laboratory Medicine, Third Affiliated Hospital of Sun Yat-sen University.

### Statistical analysis

All statistical analyses were conducted using SPSS 19.0 statistical software package and GraphPad Prism 5.0 software package (GraphPad Software, Inc., San Diego, CA, USA). Student’s *t*-test was used to compare the difference between the two groups. All bars represent the mean ± SD derived from three independent experiments. *P* values < 0.05 were statistically significant. significance levels were denoted as follows: *p < 0.05, **p < 0.01, ***p < 0.001, and ns (not significant).

### Supplementary information

Supplementary figure 1

Supplementary figure 2

Supplementary figure 3

Supplementary figure 4

Supplementary figure 5

supplemental material of uncropped WB pictures

Supplementary Figure Legends

## Data Availability

The data supporting the findings of this study in the main text and its supplementary materials are available from the corresponding author on reasonable request.
