# Supplementary figures and images for: Synergistic antitumor efficacy of aspirin plus lenvatinib in hepatocellular carcinoma via regulating of diverse signaling pathways
Source: Cell Death Discov. 2023 Nov 16;9:416. doi: 10.1038/s41420-023-01664-y (PMC10654680; doi:10.1038/s41420-023-01664-y)

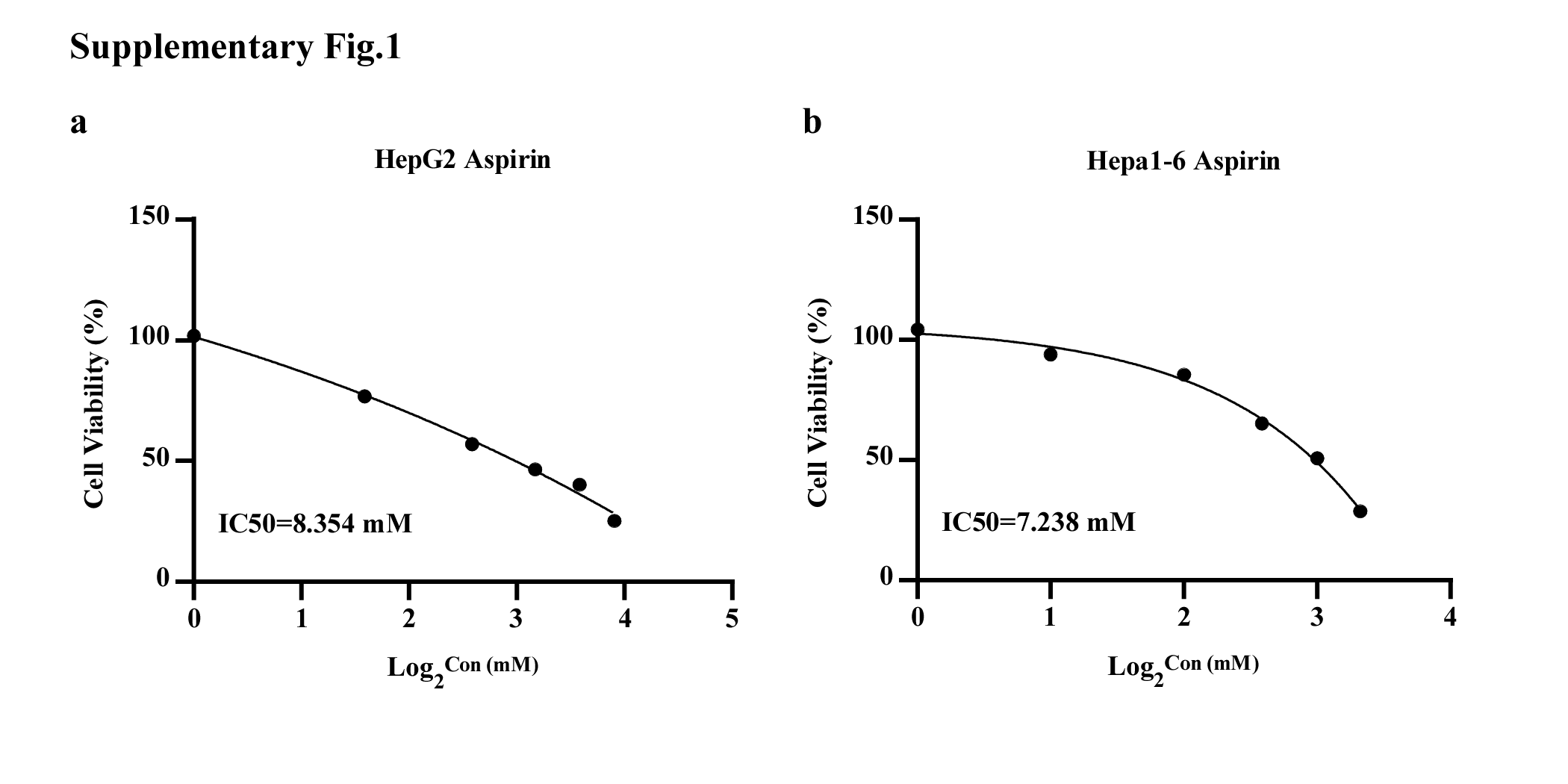

Supplement: Supplementary file 1 — Supplementary figure 1 [file 41420_2023_1664_MOESM1_ESM.tif]

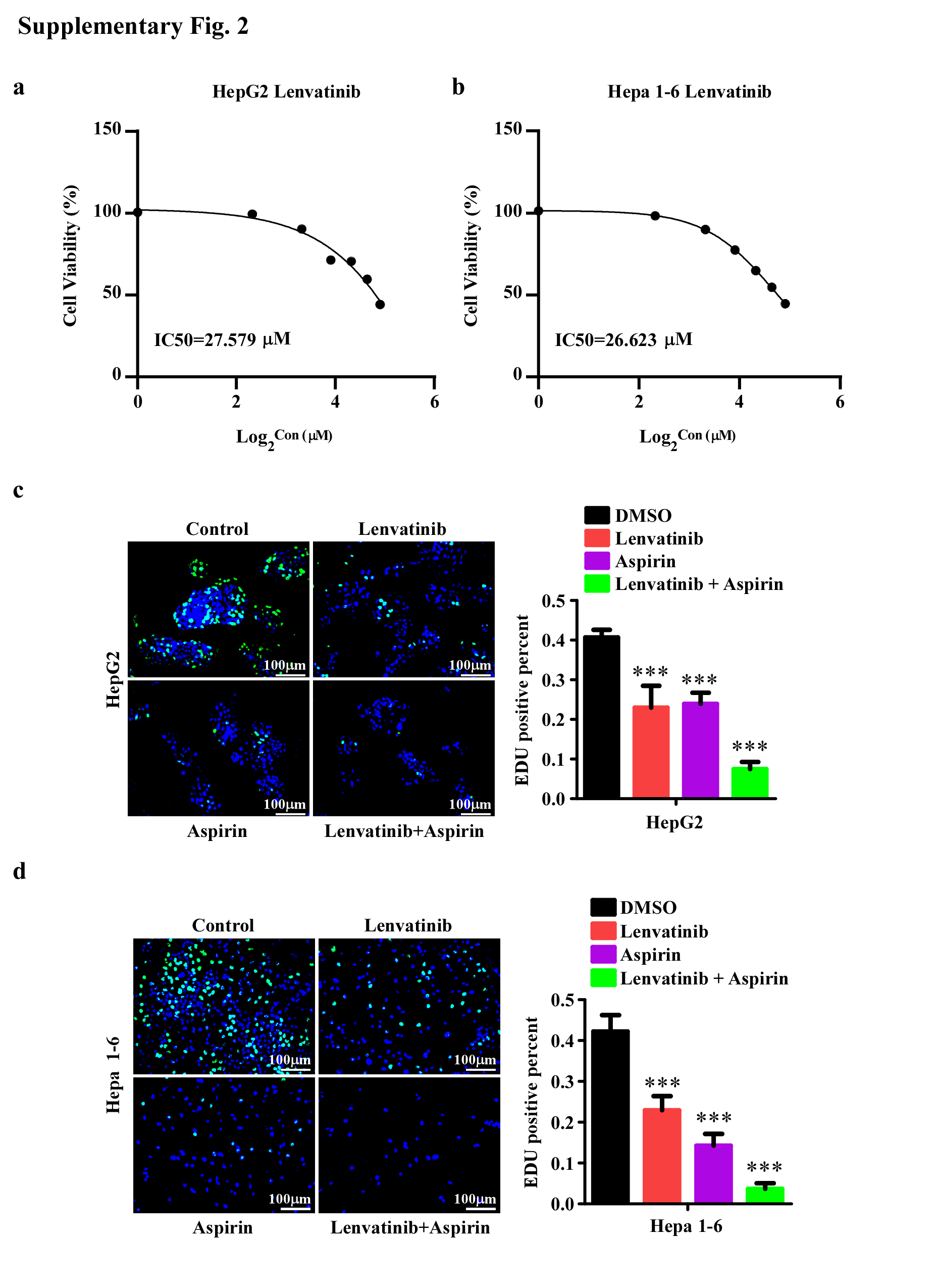

Supplement: Supplementary file 2 — Supplementary figure 2 [file 41420_2023_1664_MOESM2_ESM.tif]

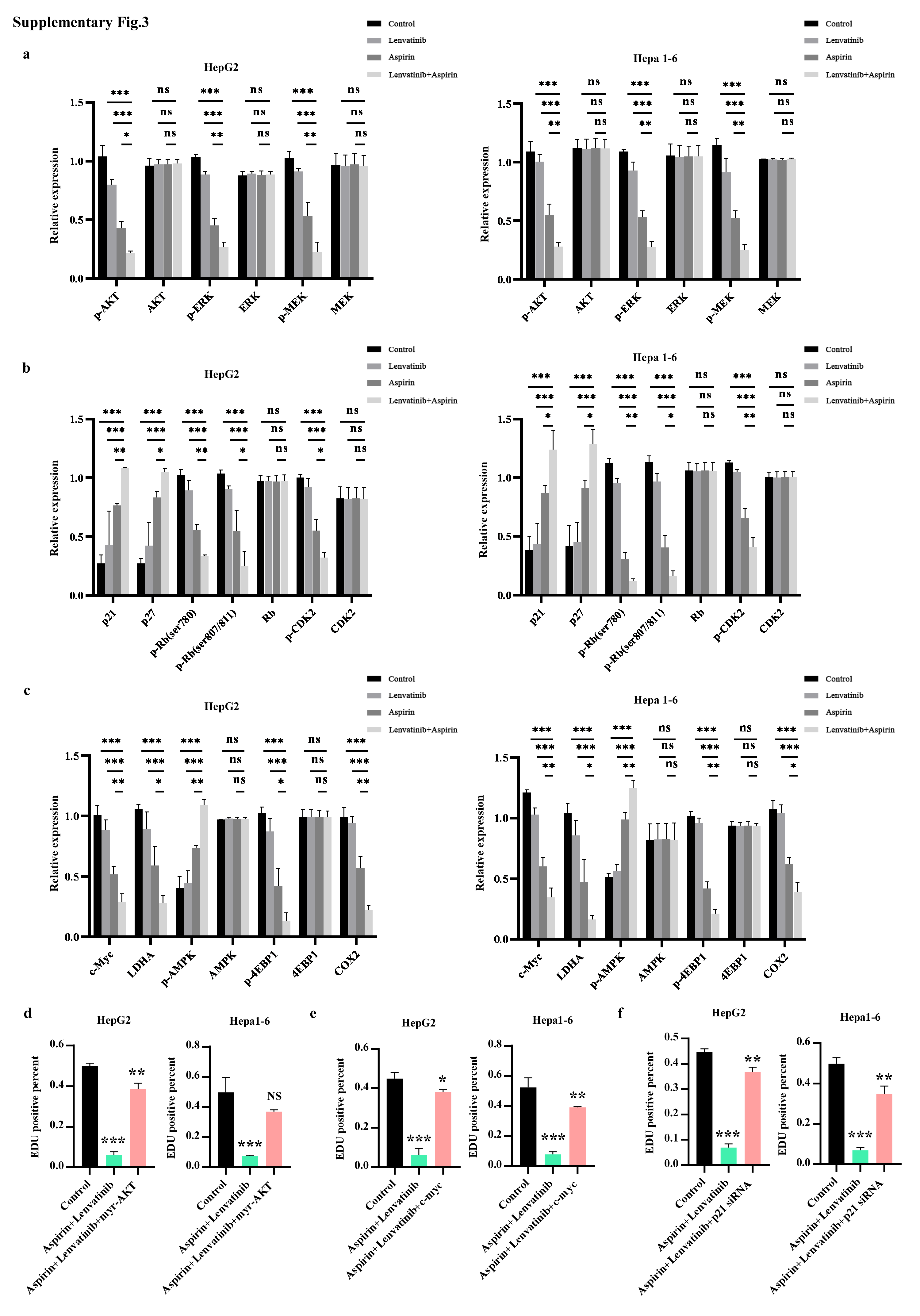

Supplement: Supplementary file 3 — Supplementary figure 3 [file 41420_2023_1664_MOESM3_ESM.tif]

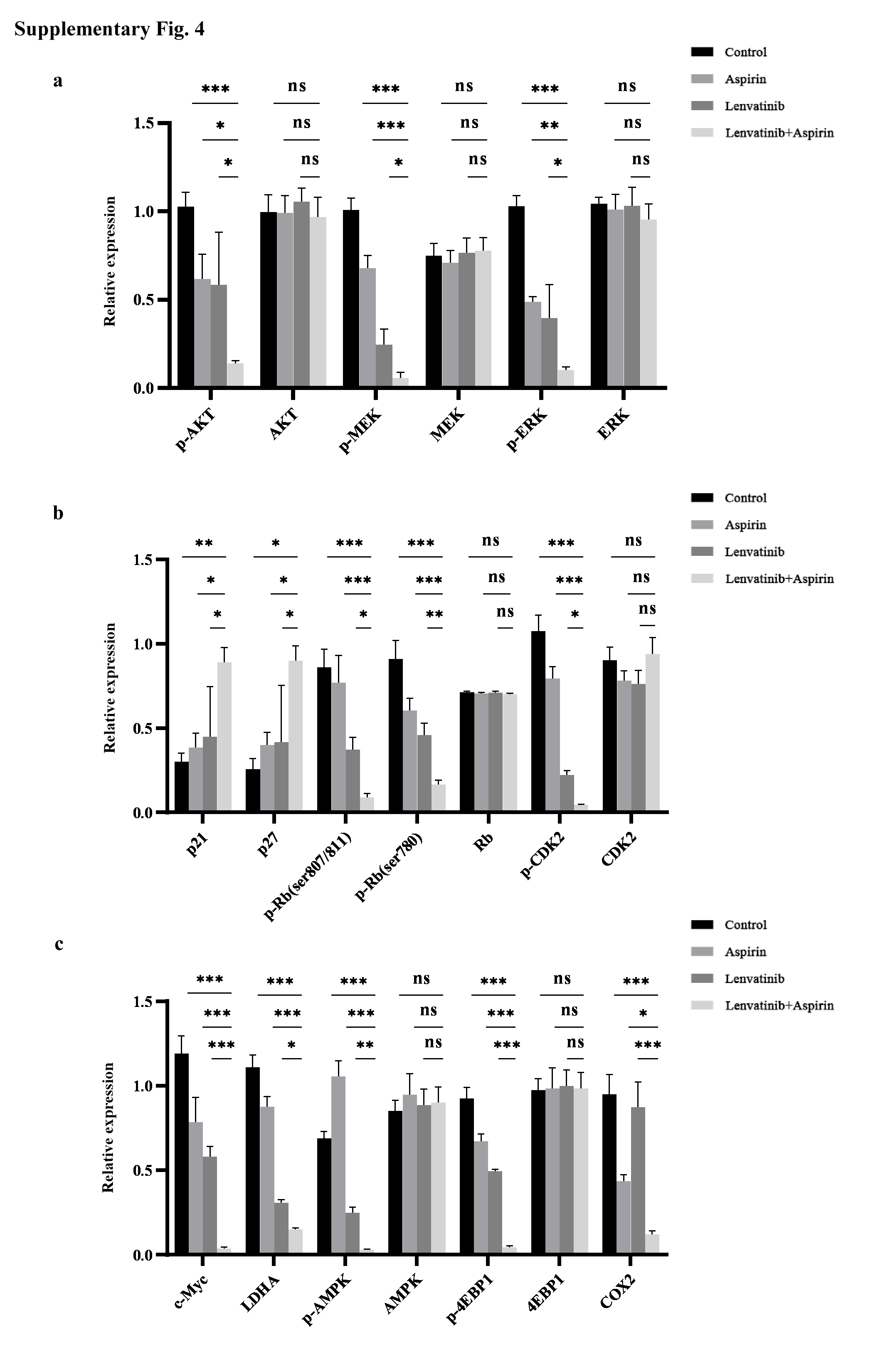

Supplement: Supplementary file 4 — Supplementary figure 4 [file 41420_2023_1664_MOESM4_ESM.tif]

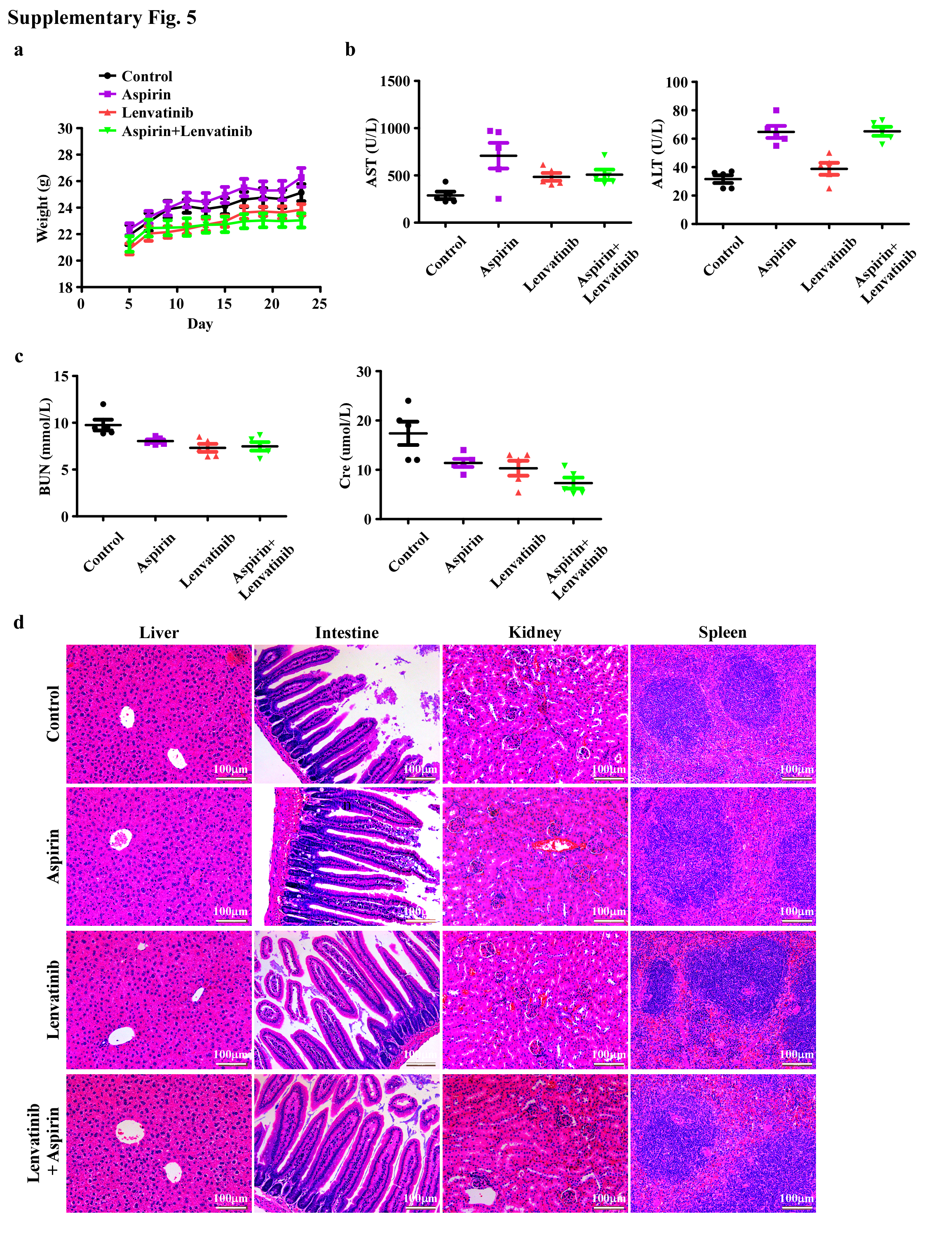

Supplement: Supplementary file 5 — Supplementary figure 5 [file 41420_2023_1664_MOESM5_ESM.tif]
