# Supplementary material for: Synergistic antitumor efficacy of aspirin plus lenvatinib in hepatocellular carcinoma via regulating of diverse signaling pathways
Source: Cell Death Discov. 2023 Nov 16;9:416. doi: 10.1038/s41420-023-01664-y (PMC10654680; doi:10.1038/s41420-023-01664-y)
Supplement: Supplementary file 6 — supplemental material of uncropped WB pictures [file 41420_2023_1664_MOESM6_ESM.docx]

Original image: Figure 2a

HepG2 p21 Hep1-6 p21







HepG2 p27 Hep1-6 p27







HepG2 P-Rb(ser780) Hep1-6 P-Rb(ser780)







HepG2 P-Rb(ser807/811) Hep1-6 P-Rb(ser807/811)







HepG2 Rb Hep1-6 Rb







HepG2 P-CDK2 Hep1-6 P-CDK2







HepG2 CDK2 Hep1-6 CDK2







HepG2 β-actin Hep1-6 β-actin







Original image: Figure 2b

HepG2 p-AKT Hep1-6 p-AKT







HepG2 AKT Hep1-6 AKT







HepG2 p-MEK Hep1-6 p-MEK







HepG2 MEK Hep1-6 MEK







HepG2 p-ERK Hep1-6 p-ERK







HepG2 ERK Hep1-6 ERK







HepG2 β-actin Hep1-6 β-actin


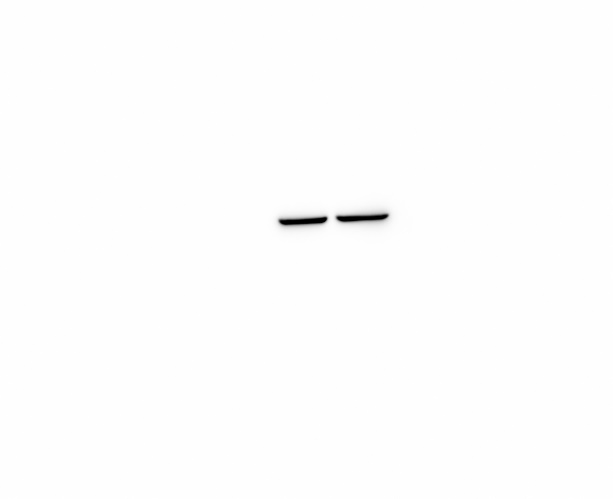

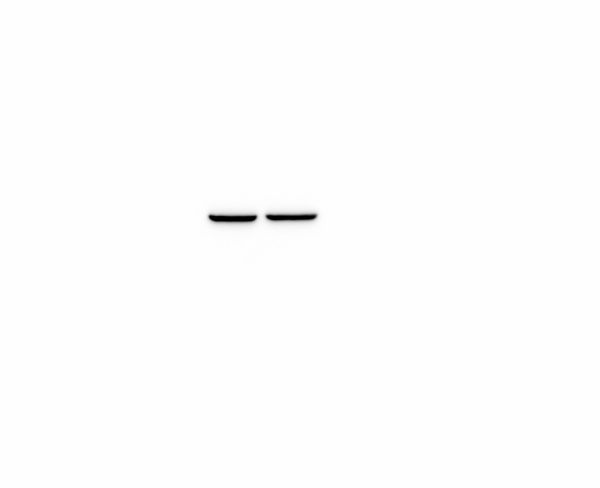


Original image: Figure 2c

HepG2 c-Myc Hep1-6 c-Myc







HepG2 PKM2 Hep1-6 PKM2







Aspirin

Control

Aspirin

Control

HepG2 LDHA Hep1-6 LDHA







HepG2 β-actin Hep1-6 β-actin







Original image: Figure 2d

HepG2 p-AMPK Hep1-6 p-AMPK







HepG2 AMPK Hep1-6 AMPK







HepG2 p-4EBP1 Hep1-6 p-4EBP1




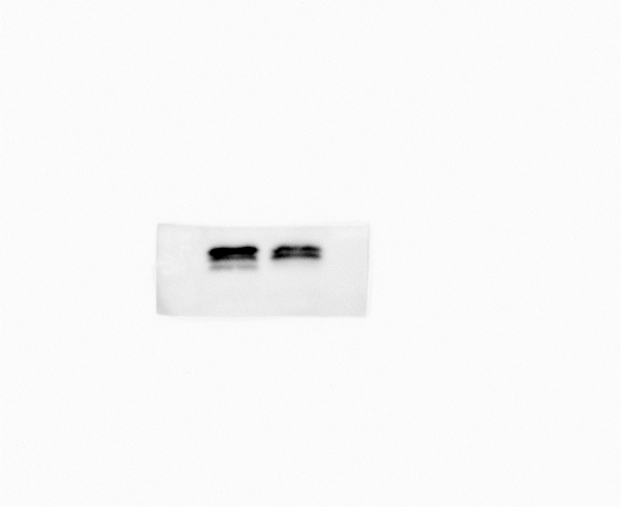


HepG2 4EBP1 Hep1-6 4EBP1







HepG2 p-P70S6K Hep1-6 p-P70S6K







HepG2 P70S6K Hep1-6 P70S6K







HepG2 β-actin Hep1-6 β-actin




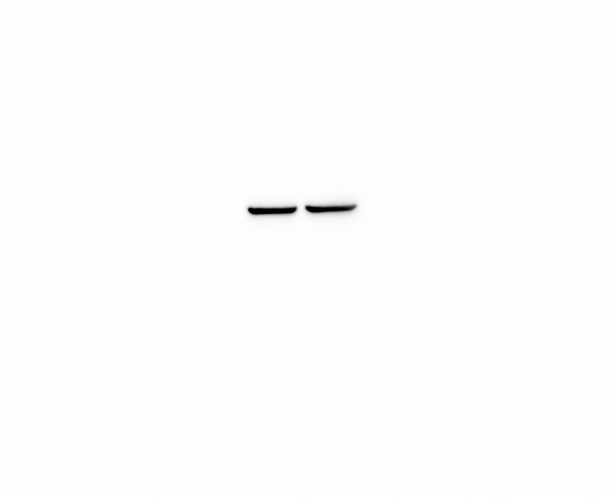


Original image: Figure 2e

HepG2 COX2 Hep1-6 COX2







HepG2 IL-1β Hep1-6 IL-1β


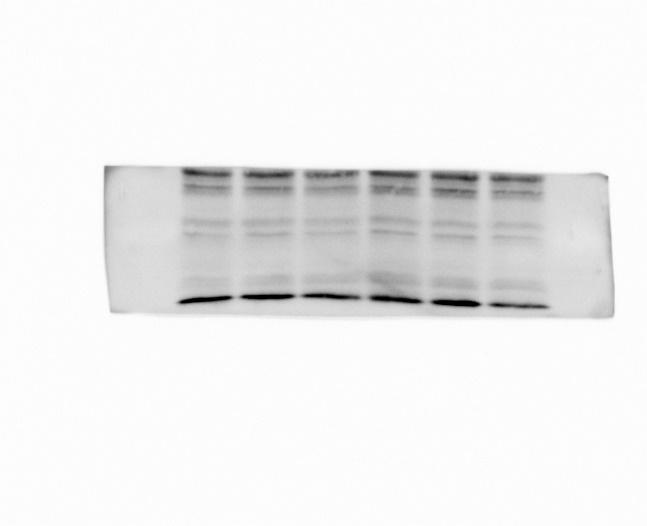

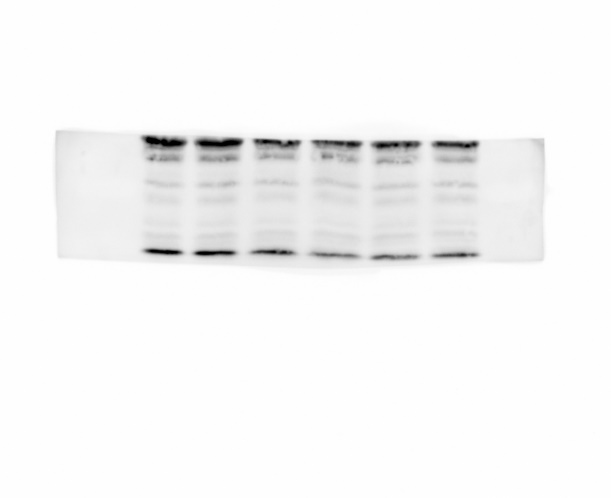


Aspirin

Control

Aspirin

Control

HepG2 β-actin Hep1-6 β-actin


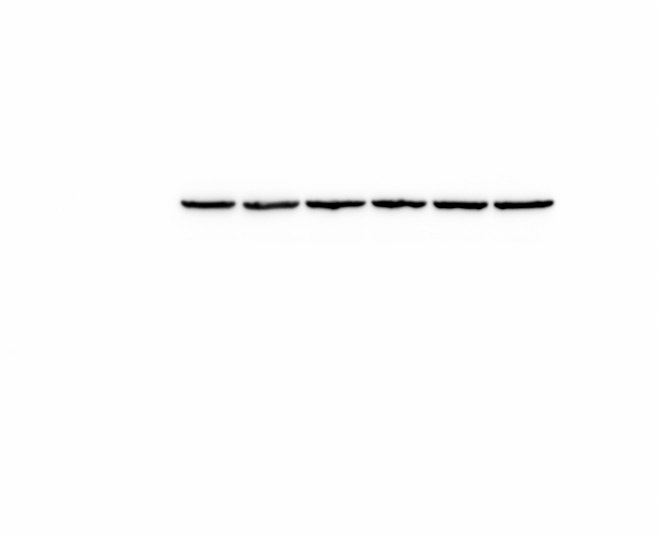




Aspirin

Control

Original image: Figure 4a

HepG2 p-AKT Hep1-6 p-AKT







HepG2 AKT Hep1-6 AKT







HepG2 p-ERK Hep 1-6 p-ERK







HepG2 ERK Hep 1-6 ERK







HepG2 p-MEK Hep1-6 p-MEK







HepG2 MEK Hep1-6 MEK







HepG2 β-actin Hep1-6 β-actin







Original image: Figure 4b

HepG2 p21 Hep1-6 p21







HepG2 p27 Hep1-6 p27







HepG2 p-Rb(ser780) Hep1-6 p-Rb(ser780)







HepG2 p-Rb(ser807/811) Hep1-6 p-Rb(ser807/811)







HepG2 Rb Hep1-6 Rb







HepG2 p-CDK2 Hep1-6 p-CDK2







HepG2 CDK2 Hep1-6 CDK2







HepG2 β-actin Hep1-6 β-actin







Original image: Figure 4c

HepG2 c-Myc Hep1-6 c-Myc







HepG2 LDHA Hep1-6 LDHA







HepG2 p-AMPK Hep1-6 p-AMPK







HepG2 AMPK Hep1-6 AMPK







HepG2 p-4EBP1 Hep1-6 p-4EBP1







HepG2 4EBP1 Hep1-6 4EBP1







HepG2 COX2 Hep1-6 COX2

HepG2 β-actin Hep1-6 β-actin

Original image: Figure 5f

p-AKT

AKT

p-MEK

MEK

p-ERK

ERK

β-actin

Original image: Figure 5g

P21

P27

p-Rb(ser807/811)

p-Rb(ser780)

Rb

p-CDK2

CDK2

β-actin

Original image: Figure 5h

c-Myc

LDHA

p-AMPK

AMPK

p-4EBP1

4EBP1

COX2

β-actin
