# Supplementary material for: Synergistic antitumor efficacy of aspirin plus lenvatinib in hepatocellular carcinoma via regulating of diverse signaling pathways
Source: Cell Death Discov. 2023 Nov 16;9:416. doi: 10.1038/s41420-023-01664-y (PMC10654680; doi:10.1038/s41420-023-01664-y)
Supplement: Supplementary file 7 — Supplementary Figure Legends [file 41420_2023_1664_MOESM7_ESM.docx]

**Supplementary Figure Legends**

**Supplementary Fig. 1** IC50 values for aspirin in cytotoxicity assays with HCC cells. (**a-b**) HepG2 (**a**) and Hepa1-6 cells (**b**) were treated with 0-10 mM concentration range of aspirin for 48h. Cell viability were detected with CCK8 assays. Cytotoxicity curves were plotted based on three independent experiments.

**Supplementary Fig. 2** Aspirin plus lenvatinib yields stronger antitumor efficacy than monotherapy. (**a-b**) IC50 values for lenvatinib in cytotoxicity assays with HCC cells. HepG2 (**a**) and Hepa1-6 cells (**b**) were treated with a 0-30 μM concentration range of lenvatinib for 48h. Cell viability was detected with CCK8 assays. Cytotoxicity curves were plotted based on three independent experiments. (**c-d**) Representative micrographs (left) and relative quantification (right) for the EdU assays of HepG2 (**c**) and Hepa1-6 (**d**) with different treatments. Error bars represent the means of three independent experiments. **P* < 0.05, ***P* < 0.01, ****P* < 0.001.

**Supplementary Fig. 3** The antitumor efficacy of drug combination depends on its regulation of various oncogenes and tumor suppressors. (**a**) Bars represent the relative protein quantification of p-AKT/AKT, p-ERK/ERK, and p-MEK/MEK of HepG2 and Hepa1-6 cells with different drug treatments. (**b**) Bars represent the relative protein quantification of p21, p27, p-Rb/Rb, and p-CDK2/CDK2 of HepG2 and Hepa1-6 cells with different drug treatments. (**c**) Bars represent the relative protein quantification of c-Myc, LDHA, p-AMPK/AMPK, p-4EBP1/4EBP1, and COX2 of HepG2 and Hepa1-6 cells with different drug treatments. (**d-f**) Bars represent the relative quantification for the EdU assays of HepG2 and Hepa1-6 cells with different treatments. Error bars represent the means of three independent experiments. **P* < 0.05, ***P* < 0.01, ****P* < 0.001. NS, no significance.

**Supplementary Fig. 4** The regulation of aspirin plus lenvatinib on the levels of multiple oncogenes and tumor suppressors *in vivo*. (**a**) Bars represent the relative protein quantification of p-AKT/AKT, p-ERK/ERK, and p-MEK/MEK in tumor tissues with different drug treatments. (**b**) Bars represent the relative protein quantification of p21, p27, p-Rb/Rb, and p-CDK2/CDK2 in tumor tissues with different drug treatments. (**c**) Bars represent the relative protein quantification of c-Myc, LDHA, p-AMPK/AMPK, p-4EBP1/4EBP1, and COX2 in tumor tissues with different drug treatments. Error bars represent the means of three independent experiments. *P < 0.05, **P < 0.01, ***P < 0.001. NS, no significance.

**Supplementary Fig. 5** Safety evaluation of aspirin plus lenvatinib in treating HCC in a mouse model. (**a**) Curves showing the weight changes of mice treated with control, aspirin, lenvatinib, and aspirin plus lenvatinib at each time point. (**b**) Changes in plasma ALT and AST levels in mice reflecting the effects of different drugs on liver function. (**c**) Changes in plasma BUN and Cre levels in mice reflecting the effects of different drugs on kidney function. (**d**) HE staining showing the morphology of the liver, intestine, kidney, and spleen of mice with different treatments. For **a-c**, data are presented as mean ± SD (n=5 mice/group)
